# Supplementary material for: Pasta as a Source of Minerals in the Diets of Poles; Effect of Culinary Processing of Pasta on the Content of Minerals
Source: Foods. 2021 Sep 9;10(9):2131. doi: 10.3390/foods10092131 (PMC8467960; doi:10.3390/foods10092131)
Supplement: Supplementary file 1 [file foods-10-02131-s001.zip › foods-1357432-supplementary.pdf]

Table S1. Characteristic of analysed products.

Pasta type	Ingredients	Cooking time, min.	Salt amount in raw product, g per kg *	Weight of pasta before cooking (g)	Weight of pasta after cooking (g) **
Penne	Wheat flour	8	0	100	319
	Wheat flour, durum flour	9	< 0.01	100	306
	Wheat flour, turmeric	9	< 0.01	100	294
	Wholemeal wheat flour	6	0.1	100	312
	Wholemeal durum flour	7	0.1	100	318
	Wholemeal spelt flour	7	0.3	100	320
	Wholemeal durum flour	13	0.15	100	312
Ditalini	Durum flour	12	< 0.1	100	301
	Wheat flour, durum flour	10	0	100	311
	Wheat flour	5	0	100	290
	Wholemeal wheat flour, durum flour	10	0.01	100	306
	Wholemeal durum flour, wheat flour; BIO	10	0.05	100	295
Gigli	Durum flour	5	0	100	258
	Wheat flour	6	0	100	251
	Spelt flour	8	0	100	274
	Wholemeal durum flour, wholemeal barley flour, amaranth flour	7	0.2	100	258
	Wholemeal durum flour, wheat flour; BIO	5	0.5	100	250
Creste	Durum flour, wheat flour, eggs, water	11	0.04	100	305
	Wheat flour	5	0	100	303
	Durum flour	12	0	100	300
	Wheat flour, durum flour, eggs	8	0.07	100	314
Fusilli	Rice flour, corn starch, tapioca starch	7	0.02	100	321
	Wheat flour	6	0	100	320
	Wheat flour	9	0	100	314
	Wholemeal wheat flour	7	0.01	100	313
	Wholemeal spelt flour	9	< 0.01	100	334
	Wholemeal durum flour	9	0.5	100	321
	Wholemeal wheat flour	4	0.011	100	321
Filini	Wheat flour, eggs, turmeric	3	0.09	100	255
	Spelt flour, turmeric	5	0.015	100	263
	Spelt flour; BIO	5	0.015	100	265
	Spelt flour	4	0	100	264
	Wholemeal spelt flour	4	0.1	100	261
	Wholemeal spelt flour; BIO	8	< 0.01	100	271
	Wholemeal spelt flour; BIO	8	0.01	100	251

* Values declared by producer; *** Mean values

Tabela S2. RDA and AI values for minerals according to Polish standards, mg.

	Na	K	Ca	Mg	Zn	Cu	Fe	Mn
	**AI**	**AI**	**RDA**	**RDA**	**RDA**	**RDA**	**RDA**	**AI**
Adults	1500 (W, M)	3500 (W, M)	1200 (W) 1000 (M)	320 (W) 420 (M)	8 (W) 11 (M)	0.9 (W, M)	18 (W) 10 (M)	1.8 (W) 2.3 (M)
Children, age 1–3	750 (W, M)	800 (W, M)	700 (W, M)	80 (W, M)	3 (W, M)	0.3 (W, M)	7 (W, M)	1.2 (W, M)
Children, age 4–6	1000 (W, M)	1100 (W, M)	1000 (W, M)	130 (W, M)	5 (W, M)	0.4 (W, M)	10 (W, M)	1.5 (W, M)
Children, age 7–12	1300 (W, M)	2400 (W, M)	1300 (W, M)	240 (W, M)	8 (W, M)	0.7 (W, M)	10 (W, M)	1.6 (W) 1.9 (M)
Children, age 13–15	1500 (W, M)	3000 (W, M)	1300 (W, M)	360 (W) 410 (M)	9 (W) 11 (M)	0.9 (W, M)	15 (W) 12 (M)	1.6 (W) 2.2 (M)
Children, age 16–18	1500 (W, M)	3500 (W, M)	1300 (W, M)	360 (W) 410 (M)	9 (W) 11 (M)	0.9 (W, M)	15 (W) 12 (M)	1.6 (W) 2.2 (M)

Table S3. Pearson correlation coefficients for NaCl+NR.

	Na	K	Ca	Mg	Zn	Cu	Fe	Mn
Na	1.000	-0.403	0.729	0.291	-0.088	-0.622	-0.165	-0.674
K	-0.403	1.000	-0.218	0.723	0.662	-0.175	-0.499	0.827
Ca	0.729	-0.218	1.000	0.380	0.205	-0.547	-0.513	-0.357
Mg	0.291	0.723	0.380	1.000	0.783	-0.510	-0.790	0.288
Zn	-0.088	0.662	0.205	0.783	1.000	-0.136	-0.732	0.322
Cu	-0.622	-0.175	-0.547	-0.510	-0.136	1.000	0.068	-0.101
Fe	-0.165	-0.499	-0.513	-0.790	-0.732	0.068	1.000	-0.107
Mn	-0.674	0.827	-0.357	0.288	0.322	-0.101	-0.107	1.000

value *r*; strong correlations are marked in red; NaCl + NR – cooked with salt and not rinsed

Table S4. Pearson correlation coefficients for NaCl+R.

	**Na**	**K**	**Ca**	**Mg**	**Zn**	**Cu**	**Fe**	**Mn**
Na	1.000	-0.403	0.748	0.129	-0.117	-0.571	-0.302	-0.684
K	-0.403	1.000	-0.413	0.803	0.927	-0.096	-0.506	0.374
Ca	0.748	-0.413	1.000	0.121	-0.088	-0.541	-0.411	-0.517
Mg	0.129	0.803	0.121	1.000	0.953	-0.345	-0.882	-0.041
Zn	-0.117	0.927	-0.088	0.953	1.000	-0.330	-0.722	0.242
Cu	-0.571	-0.096	-0.541	-0.345	-0.330	1.000	0.178	-0.119
Fe	-0.302	-0.506	-0.411	-0.882	-0.722	0.178	1.000	0.402
Mn	-0.684	0.374	-0.517	-0.041	0.242	-0.119	0.402	1.000

value *r*; strong correlations are marked in red; NaCl + R – cooked with salt and rinsed

Table S5. Pearson correlation coefficients for NR.

	Na	K	Ca	Mg	Zn	Cu	Fe	Mn
Na	1.000	0.591	0.757	0.284	0.613	-0.483	-0.492	0.023
K	0.591	1.000	0.577	0.400	0.536	-0.618	-0.380	0.747
Ca	0.757	0.577	1.000	0.440	0.538	-0.452	-0.172	0.012
Mg	0.284	0.400	0.440	1.000	0.824	-0.266	-0.639	0.102
Zn	0.613	0.536	0.538	0.824	1.000	-0.093	-0.901	0.243
Cu	-0.483	-0.618	-0.452	-0.266	-0.093	1.000	-0.078	-0.130
Fe	-0.492	-0.380	-0.172	-0.639	-0.901	-0.078	1.000	-0.267
Mn	0.023	0.747	0.012	0.102	0.243	-0.130	-0.267	1.000

value *r*; strong correlations are marked in red; NR – cooked without salt and not rinsed

Table S6. Pearson correlation coefficients for R.

	Na	K	Ca	Mg	Zn	Cu	Fe	Mn
Na	1.000	0.537	0.968	0.602	0.544	-0.336	-0.525	-0.073
K	0.537	1.000	0.657	0.564	0.437	-0.602	-0.346	0.634
Ca	0.968	0.657	1.000	0.770	0.675	-0.397	-0.616	0.059
Mg	0.602	0.564	0.770	1.000	0.914	-0.190	-0.767	0.261
Zn	0.544	0.437	0.675	0.914	1.000	0.172	-0.888	0.335
Cu	-0.336	-0.602	-0.397	-0.190	0.172	1.000	-0.130	0.049
Fe	-0.525	-0.346	-0.616	-0.767	-0.888	-0.130	1.000	-0.082
Mn	-0.073	0.634	0.059	0.261	0.335	0.049	-0.082	1.000

value *r*; strong correlations are marked in red; R – cooked without salt and rinsed
